# Cell-free IgG-aggregates in plasma of patients with chronic lymphocytic leukemia cause chronic activation of the classical complement pathway

**DOI:** 10.1371/journal.pone.0230033

**Published:** 2020-03-09

**Authors:** Regina Michelis, Tamar Tadmor, Ariel Aviv, Galia Stemer, Rawan Majdob, Lev Shvidel, Mona Shehadeh, Masad Barhoum, Andrei Braester

**Affiliations:** 1 The Institute for Medical Research, Galilee Medical Center, Nahariya, Israel; 2 Hematology Division, Bnai Zion Medical Center, Haifa, Israel; 3 The Ruth and Bruce Rappaport Faculty of Medicine, Technion, Haifa, Israel; 4 Department of Hematology, Emek Medical Center, Afula, Israel; 5 Hematology Institute, Kaplan Medical Center, Rehovot, Israel; 6 Faculty of Medicine, Hebrew University, Jerusalem, Israel; 7 Biochemistry Laboratory, Galilee Medical Center, Nahariya, Israel; 8 Institute of Hematology, Galilee Medical Center, Nahariya, Israel; 9 Azrieli Faculty of Medicine, Bar Ilan University, Safed, Israel; University of Manitoba, CANADA

## Abstract

Therapy regimens for Chronic lymphocytic leukemia (CLL) commonly include chemotherapy and immunotherapy, which act through complement-mediated-cytotoxicity (CDC) and other mechanisms. CDC depends on several factors, including the availability and activity of the complement classical pathway (CP). Recently, a significant decrease in CP activity was shown to be associated with an immunoglobulin-C5a complex (Ig-C5a) and other markers of chronic CP activation in 40% of the patients. The study focused on the involvement of IgG-hexamers, an established CP activator, in the mechanism of chronic CP activation in CLL. Sera from 51 naïve CLL patients and 20 normal controls were collected. CP and alternative pathway (AP) activities were followed by the complement activity marker sC5b-9. Serum high molecular weight (HMW) proteins were collected by gel-filtration chromatography and their complement activation capacity was assessed. The levels of IgM, another established CP activator, were measured. Data were associated with the presence of Ig-C5a. Baseline levels of activation markers negatively correlated with CP and the AP activities, supporting chronic complement activation. In patients with Ig-C5a, HMW proteins that are not IgM, activated the complement. HMW proteins were identified as IgG-aggregates by affinity binding assays and Western blot analysis. The data indicate chronic CP activation, mediated by cell-free IgG-hexamers as a cause of decreased CP activity in part of the CLL population. This mechanism may affect immunotherapy outcomes due to compromised CP activity and CDC.

## Introduction

Chronic lymphocytic leukemia (CLL), the most common adult leukemia in the Western world, accounts for 30% of all leukemia cases. CLL is characterized by ≥5000 monoclonal B lymphocytes/μl in peripheral blood, which co-express the antigens CD5, CD19, CD20 and CD23 [1], and exhibit mature phenotype. The lymphocytes accumulate in peripheral blood, bone marrow, spleen, and secondary lymphoid organs. CLL is a heterogenous disease, with a survival that depends on several factors, including the genomic landscape of CLL, with long known (such as del[13q14], del[17p], del[11q] and others) and more recently described (NOTCH1, MYD88, TP53, ATM, SF3B1, FBXW7, POT1, CHD2, RPS15 and others) mutations [1]. CLL is also associated with an inherent immune dysfunction that is related to morbidity and mortality as well as to infections, which account for 50 to 60% of all deaths [1,2]. The infections in CLL are related both to the disease and to the immuno-suppressive effects of the therapy. The therapeutic approach in fit CLL patients includes therapeutic monoclonal antibodies (mAbs), commonly used in combination with chemotherapy [1–3], so that the mAb drugs trigger immune responses against the leukemic B-cells that synergize with cytotoxic chemotherapeutic agents. The most clinically used immunotherapeutic drugs are Rituximab (RTX), Obinutuzumab, Ofatumumab (which target the CD20) and Alemtuzumab (anti CD52), expressed on B-CLL cell surface [4,5]. The anti-tumor effects of the mAbs are mediated through several distinct mechanisms: complement-dependent cytotoxicity (CDC), antibody-dependent cell-mediated cytotoxicity (ADCC), lysosomal-dependent cell death (lysosome membrane permeability—LMP) and phagocytosis [4–6]. Thus, the efficacy of the therapeutic mAbs depends on various factors, including the availability and activity of the complement (C) system [7–9].

We have recently demonstrated a subgroup of CLL patients with increased levels of C activation markers and an Ig-C5a complex, and decreased activity of the classical C pathway (CP), suggesting chronic CP activation [10]. The CP is a main activation cascade of the C system, which also includes the alternative (AP) and lectin pathways. The CP is activated by antigen-antibody complexes, assembled from IgM or IgG, which bind to the C1 component of the CP and initiate the CP cascade. The lectin pathway is triggered by mannan-binding lectin (MBL) or ficolins bound to carbohydrates and other pathogen-associated molecules and the AP may be triggered directly by foreign substances such as microorganisms or artificial biomaterials. Although the C pathways differ in their stimulators, they all converge in a common pathway, activating the assembly of the membrane attack complex (MAC, C5b-9). The C5b-9 elicits cell lysis by inserting itself into the lipid bilayer of the pathogen's cell membranes [11,12].

In CLL, IgG and IgM levels are either normal or reduced, but not increased [13–15]. IgG in its monomeric form cannot activate the CP and IgG-dependent activation of the CP depends on formation of HMW IgG aggregates, particularly in the form of hexamers (IgG_6_), which are formed via Fc:Fc interactions [16]. The steps related to the initiation of the C cascade were described on cell surface [16,17], and cell-free IgG_6_ in plasma have not been reported yet.

In this study we examined the presence of cell-free IgG_6_ in plasma, as a potential cause for constant CP activation in part of the CLL population.

## Material and methods

### Subjects

Blood samples were collected from 51 naïve CLL patients and 20 normal controls (NC). Plasma and sera were separated immediately and frozen at -80°C. Samples were carefully handled as described [18] in order to avoid spontaneous C activation. Biochemical and hematological parameters, and CLL staging were recorded. The levels of C3 and C4 were quantified using an immunoturbidimetric test (ABBOTT laboratories, USA) on the ARCHITECT clinical chemistry analyzer. The study was approved by the Helsinki Committee (Institutional Review Board) of Galilee Medical Center, Nahariya, Israel, in compliance with the declaration of Helsinki, and all subjects signed a written informed consent form.

### Analysis of Ig-C5a

Plasma samples were subjected to albumin and Ig depletion using a commercial kit (Sigma). The isoforms of C5 were studied under non-reducing conditions in the samples by Western blot analysis, using mouse monoclonal anti-human C5 antibodies (Quidel, AB_452484) and anti-human C5a antibodies (Complement Technology, A221).

### Complement activity

C activation was followed by the levels of soluble C5b-9 (sC5b-9, also named terminal C complex—TCC or MAC), using a commercial kit (Quidel, A029). In some experiments, the C activation marker C5a was assessed by a commercial ELISA kit (RayBiotech, ELH-CCC5a). The levels of sC5b-9 were determined in sera before (Basal sC5b-9 levels) and after in-vitro activation induced via the CP or AP, using aggregated IgG or Zymosan, respectively. Activations were performed in DGVB^2+^ buffer, as described [10]. DGVB^2+^ buffer: 1 mM MgCl_2_, 0.15 mM CaCl_2_, 71 mM NaCl, 0.1% (w/v) gelatin, 2.5% (w/v) dextrose, and 2.47 mM sodium 5′,5′′-diethyl barbiturate (pH 7.35). The C activation capacity of the fractions collected by gel-filtration chromatography was also assessed by using normal C system (normal subjects’ serum). For activation, 1 μg from each collected fraction was diluted to 20 μl in column buffer and mixed with 80 μl DGVB^2+^ buffer and 100 μl diluted normal serum (5% serum diluted in DGVB^2+^ buffer) to give a final concentration of 5 μg/ml for the fraction's proteins and a final serum concentration of 2.5%. Samples were incubated at 37°C for 45 min (or 2 hours in the experiments with gel-filtration chromatography fractions) and the reaction was stopped as described [10]. In some experiments C1q-depleted serum (Sigma) was used in a concentration of 2.5%, with and without C1q supplementation. In these experiments purified C1q (Sigma) was used in a final concentration of 1μg C1q/1 ml serum. Samples were incubated at 37°C for 2 hours and the reaction was then stopped as described [10]. Zymosan and in-vitro aggregated IgG, which are effective activators of the AP and CP, respectively, were used as controls. C1q-depleted serum that was incubated only with C1q but without HMW fractions, was used as a negative control for each experiment. The change in sC5b-9 levels due to C1q supplementation is presented as Delta (Δ) relative to non-supplemented sera, and after subtraction of the negative control values.

### Assay of the complement activation pathways

Identification of the activated C pathways was performed using the ELISA based Total Complement Functional Screen®-kit (Wieslab, 36-COMPL-300) according to the manufacturer's instructions. In principle, the negative and positive kit controls are given the values of 0% and 100%, respectively, and refer to the activity in the sample, thus 100% indicates no previous activation. The values of the sera are expressed as percentages of the positive control.

### Gel filtration chromatography of serum proteins

All chemicals were obtained from SIGMA (St. Louis, MO, USA). To obtain fractions with various molecular weights, serum samples were subjected to gel filtration chromatography as described [19] with minor modifications: A 7 ml column of Sephacryl S-200-HR was prepared and the column was washed with 3 volumes of column buffer (50 mM Tris, 100 mM NaCl, pH 7.5). Two hundred μl of plasma or serum were diluted with Tris buffer to a final concentration of 50 mM Tris at pH 7.5, centrifuged for 3 min at 8,000g and the supernatant was loaded on the column. Proteins were eluted in column buffer in 0.5 ml fractions. The early eluting fraction was named high molecular weight (HMW) fraction, the albumin containing fractions was collected after 3 ml and the last eluting fraction was named low molecular weight (LMW) fraction. The HMW and LMW fractions were acetone precipitated, the pellets were dissolved in column buffer and protein concentration was measured (by Nanodrop) prior to further analyses.

### IgM analysis

IgM levels were measured in the HMW fractions after their addition to the diluted normal serum, by a commercial ELISA kit (affymetrix eBioscience, BMS2098).

### IgG analysis in the HMW fractions

HMW fractions from patients and NC were studied by Western blot analysis under reducing conditions, using rabbit polyclonal anti-human IgG antibodies (Sigma, SAB3701322). The presence of IgG in the HMW fractions was also studied by IgG depletion. HMW fractions were subjected to IgG depletion using a commercial kit (Sigma, Albumin and IgG depletion kit, PROTBA-1KT). The HMW fractions with and without IgG depletion were used for activity analysis as detailed above (section "4.3 Complement activity") and the change in activity was calculated as percentage of the activity measured in the non-depleted fractions.

### Statistical analysis

Data parameters were analyzed by unpaired t-test, by linear regression analysis and by Wilcoxon Signed Ranks Test, as appropriate. P<0.05 was considered significant.

## Results

### Characteristics of the subjects' groups

The characteristics of the NCgroup, CLL patients and the patients' subgroups, with undetectable or detectable Ig-C5a, are given in Table 1. The levels of C3 were significantly higher (p<0.002) in CLL patients compared to NC, as previously observed [10]. More than 80% of the patients had C3 levels within the normal range (82–185 and 83-193mg/dl for males and females, respectively, Table 1). When patients were divided according to the detection of Ig-C5a, C3 levels were found to be significantly higher (compared to NC) in CLL patients with detectable Ig-C5a, as observed previously [10]. C4 levels in the patients and in the sub-groups were similar to NC. The majority of patients (96%) had C4 levels within the normal range (15–53 and 15-57mg/dl for males and females, respectively). All disease parameters including stage, lymphocyte counts and the levels of IgA, IgG and IgM did not differ significantly between the CLL sub-groups.

**Table 1:** Characteristics of the study population.

	NC	CLL	CLL Undetectable Ig-C5a	CLL Detectable Ig-C5a
**n**	20	51	25	26
**Gender (male/female)**	9/9	32/19	15/10	17/9
**Age (Years)**	56±2*	67±3	68±3	65±2
**Binet Stage (A/B/C)**	-	36/8/7	18/4/3	18/4/4
**[%A/%B/%C]**		[70/16/14]	[72/16/12]	[69/15/15]
**Serum C3 (mg/dl)**	120.2±5	147±5^#^	138±7	160±8^#^
**Serum C4 (mg/ dl)**	28±2	30±2	28±2	33±2
**Lymphocytes count (x10**^**3**^**/**μ**l)**	2.5±0.2^§^	24.9±6.5	30.3±10.1	16.3±4.9
**IgA (mg/dL)**	nd	128±18	142±30	119±23
**IgG (mg/dL)**	nd	965±74	1035±151	920±79
**IgM (mg/dL)**	nd	57±9	56±8	57±14

All values are given as mean±SEM.

* indicates significant p value (p<0.001) compared to each other group.

# indicates significant (p<0.002) value compared to the NC group.

§ indicates significant p value (p<0.05) compared to each other group. nd–not determined.

### Levels of complement activity markers before and after activation

The levels of the C activity marker sC5b-9, were measured at baseline (without activation) and after activation of the CP and AP. In patients with detectable Ig-C5a, the levels of sC5b-9 after CP activation were significantly reduced, while basal levels were significantly increased compared to the other subjects’ groups (NC and patients with undetected Ig-C5a, Table 2). These data may represent the exhaustion of the CP as a result of previous CP activation, occurring for a prolonged period or chronically.

**Table 2:** Characteristics of C activity in the study population.

		NC	CLL	CLL undetectable Ig-C5a	CLL detectable Ig-C5a
**n**		14	39	23	16
**basal sC5b-9 (ng/ml)**		652±130*	3103±459	2228±554	4104±706
**CP**	**Activity (sC5b-9, ng/ml)**	22070±1955	15124±1706^#^	16792±2400	12672±2258^#^
	**Correlation with basal sC5b-9**	p = 0.061	P = 0.001	p = 0.032	p = 0.022
**AP**	**Activity (sC5b-9, ng/ml)**	62965±2790	53261±3901	54410±5037	51665±6310
	**Correlation with basal sC5b-9**	p = 0.005	p = 0.026	p = 0.302	p = 0.042

All values are given as mean±SEM.

* indicates significance (p≤0.03) compared to each of the other groups.

# indicates significance (p≤0.03) compared to the NC group.

**Fig 1:**
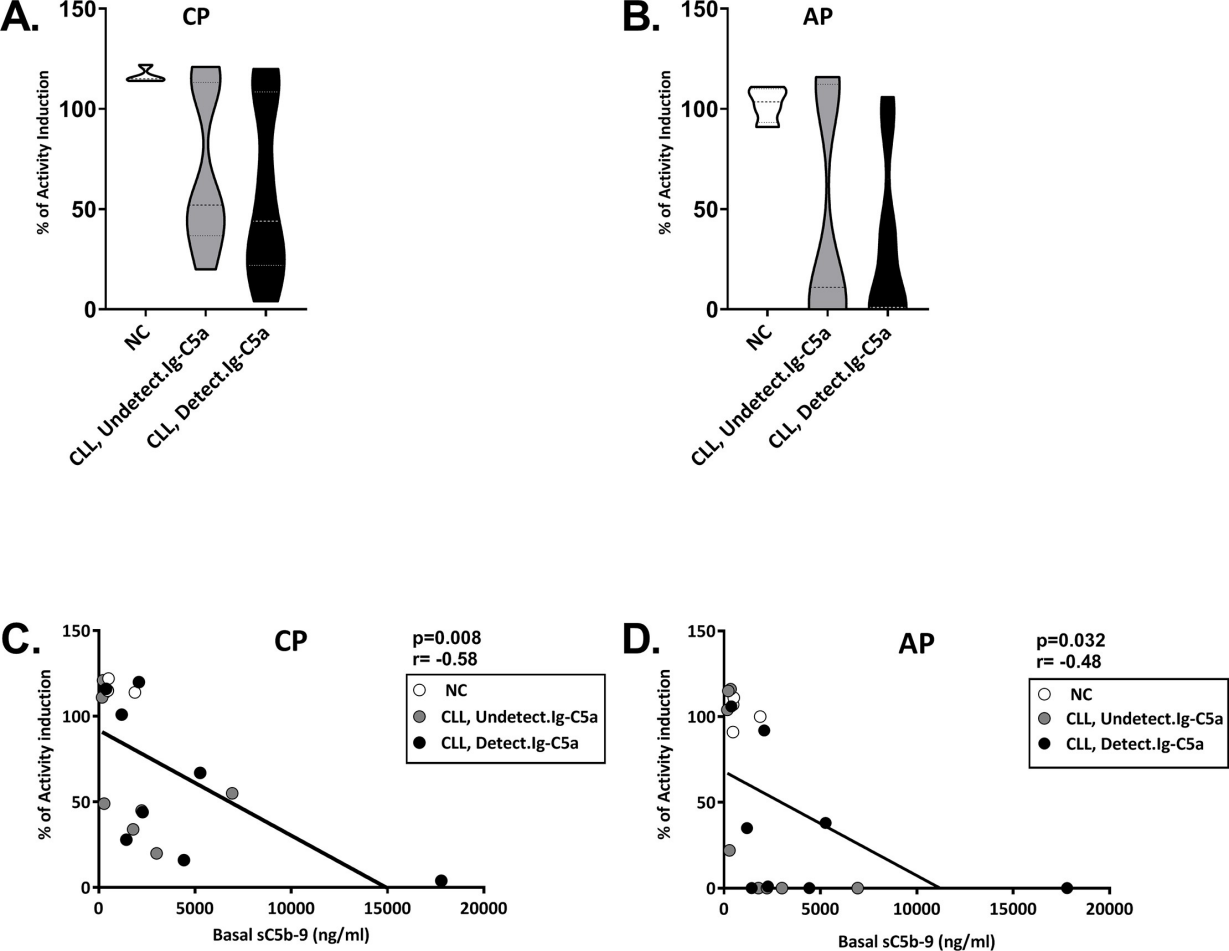
Complement pathway screening. A pathway screening kit was used to indicate previous activation via various C pathways in the subjects' sera (A,B). The values of the sera are expressed as percentage of the positive control, so that 100% indicates no previous activation. The data obtained was correlated with basal sC5b-9 levels (C,D). NC (white circles): n = 4; CLL with undetectable Ig-C5a (grey circles): n = 8; CLL with detectable Ig-C5a (black circles): n = 8.

This previous or chronic activation of the CP (and the AP) was studied by using a pathway screening kit (Fig 1). The data obtained by the kit indicated previous activation of the CP (and AP, Fig 1A and 1B respectively) and negative correlation with basal sC5b-9 levels (Fig 1C and 1D).

**Fig 2:**
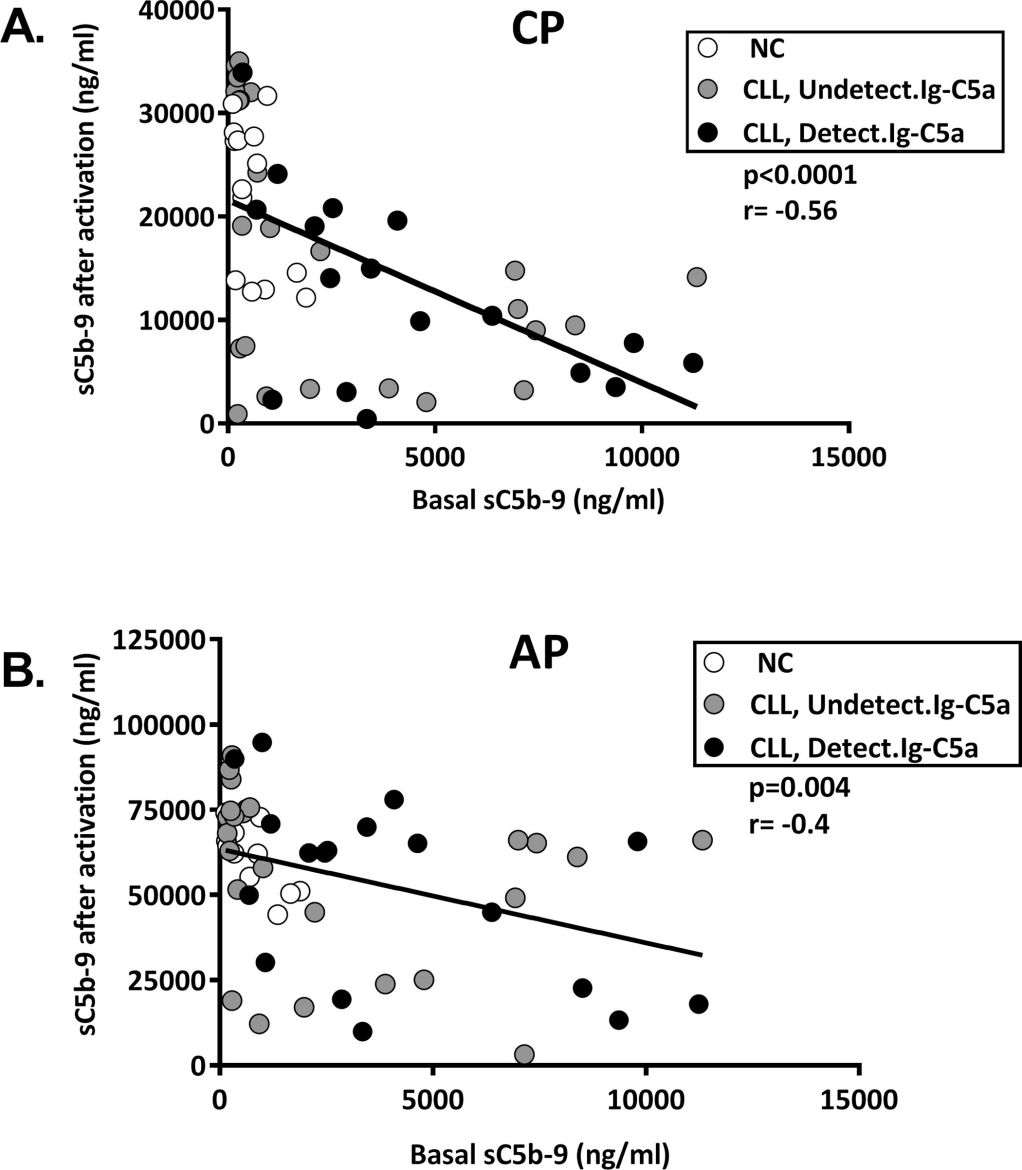
Correlation of complement activity with basal activation marker. C was activated in-vitro via the CP (by aggregated IgG) or via the AP (by Zymosan) and the levels of sC5b-9 were quantified. Basal sC5b-9 levels were subtracted. The correlations between sC5b-9 levels at baseline and after activations of the CP (**A**) or the AP (**B**) were analyzed in all subjects. NC (white circles): n = 13; CLL with undetectable Ig-C5a (grey circles): n = 23; CLL with detectable Ig-C5a (black circles): n = 16.

As these data were obtained by the kit, which is semi-quantitative (according to the manufacturer), we confirmed and expanded the data by examining the correlations between sC5b-9 levels at baseline and after C activation (Fig 2). The sC5b-9 levels after CP and AP activation showed significantly inverse correlations with basal sC5b-9 values (Fig 2A and 2B), thus supporting chronic C activation.

The correlation data between sC5b-9 levels at baseline and after activation were more conclusive in the CP than in AP, showing significant negative correlations in the entire CLL cohort as well as in the CLL subgroups (Table 2). For the AP, significant negative correlations were found only in the entire patient cohort and in patients with detectable Ig-C5a, but not in patients with undetectable Ig-C5a (Table 2). Moreover, all the p values for CP correlations in the patient groups were lower than the values for AP (Table 2).

### High molecular weight proteins from patients' serum act as complement activators

The C exhaustion in patients with detectable Ig-C5a may have occurred due to the persistent occurrence of C activators. Unlike the AP, the CP has only a limited number of activators, lacks the self-enhancing mechanism (no amplification loop), and has relevance for future immunotherapies. Thus, we searched for a CP activator in the serum of these patients.

**Fig 3:**
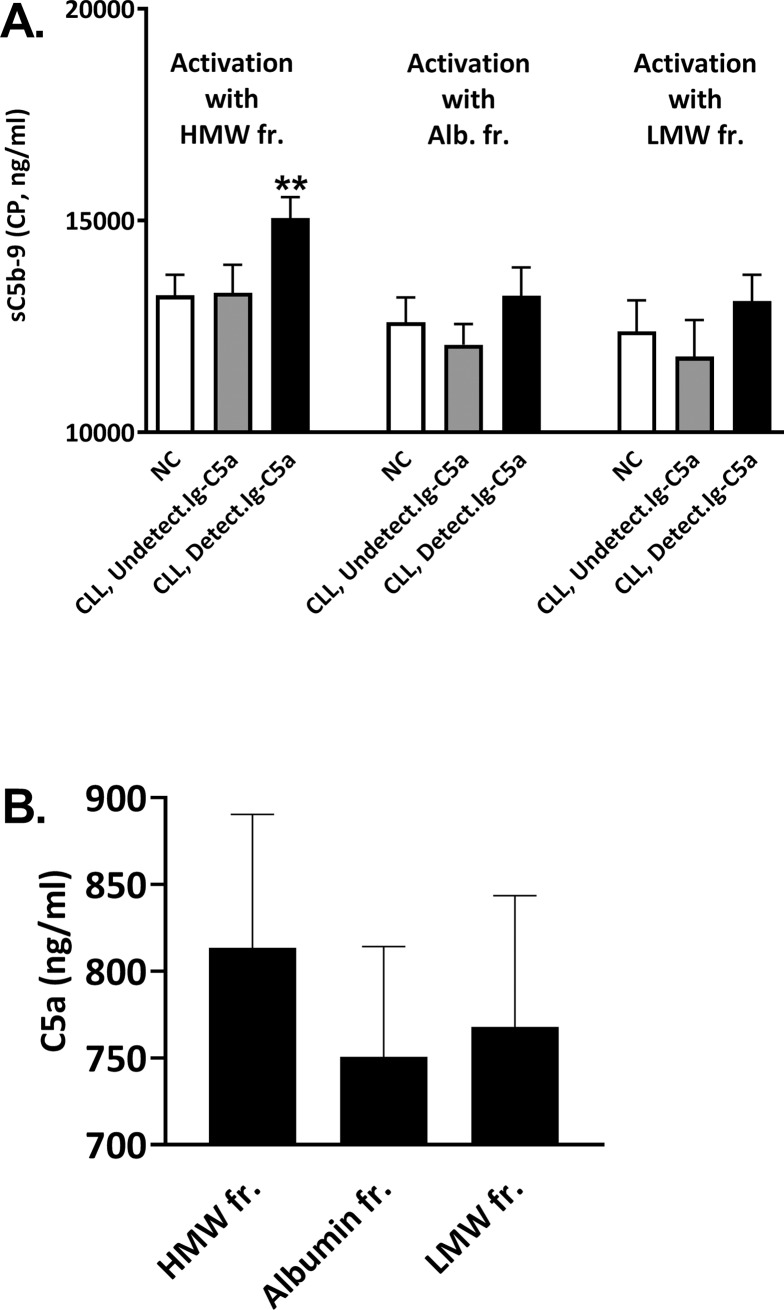
Activation of complement by the serum-fractions. Sera from CLL patients and NC were subjected to gel filtration chromatography. HMW-, albumin- and LMW-fractions were collected and used for activation of the C system in normal serum. The C activation marker sC5b-9 was measured (**A**). NC (white bars): n = 15; CLL with undetectable Ig-C5a (grey bars): n = 13; CLL with detectable Ig-C5a (black bars): n = 15. In some experiments where fractions from patients with detectable Ig-C5a were used (n = 6), the C activation marker C5a was measured (**B**).

The most common CP activators are high molecular weight (HMW) immunoglobulins, isotypes IgM, and IgG in the form of IgG-aggregates. HMW protein fractions were separated from patients' serum using gel-filtration chromatography, and their ability to activate C was measured and compared with albumin containing fractions and with low molecular weight (LMW) protein fractions (Fig 3). The results indicated that HMW fractions from patients with detectable Ig-C5a were able to activate the C more than HMW from the other subjects’ groups (NC and patients with undetected Ig-C5a), and also more than albumin or LMW fractions from any other group (Fig 3A). In the HMW trials, we used the levels of sC5b-9 to follow C activity. However, given that the sC5b-9 levels are increased in patients with detected Ig-C5a [10] and that its MW is high, we examined the possibility that the measured sC5b-9 originates from the HMW fractions rather than from de-novo activation of the C. To test this potential artifact, the levels of C5a, another activation product but with a low MW (11kD), was performed. The C5a analysis, performed in sera samples that were activated by the fractions from patients with detectable Ig-C5a, showed similar results to the sC5b-9 results: the HMW fractions activated the C significantly more than albumin or LMW fractions (Fig 3B).

### Identification of the pathway activated by the HMW fractions

**Fig 4:**
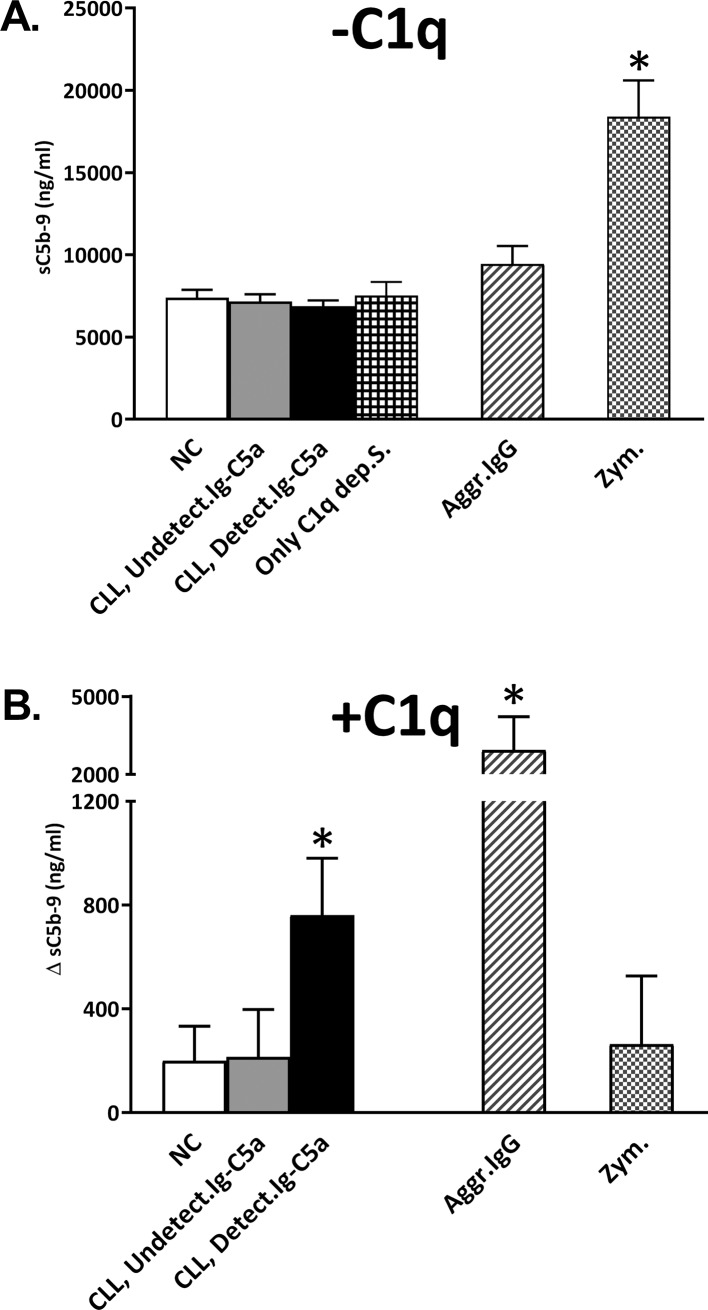
CP activation by the serum HMW-fractions. HMW fractions separated from CLL patients and NC were added to C1q-depleted serum and C activity was measured (by sC5b-9 levels) without (**A**) and with (**B**) C1q supplementation. Zymosan and in-vitro aggregated IgG were used as controls, being effective activators of the AP and CP, respectively. The change in sC5b-9 levels due to C1q supplementation is presented as Delta (Δ) relative to non-supplemented sera. C1q-depleted serum incubated with: NC HMW fractions (white bars): n = 10; HMW fractions from CLL with undetectable Ig-C5a (grey bars): n = 10; HMW fractions from CLL with detectable Ig-C5a (black bars): n = 11; only C1q-depleted serum without HMW fractions (n = 4); in-vitro aggregated IgG ▨ (n = 3); zymosan (n = 5). *p<0.01 vs. all.

HMW fractions from patients with detectable Ig-C5a caused C activation. As all the pathways produce the same C activity markers, these markers cannot assist in identifying the pathway which was activated. We anticipated that the HMW fractions activate the CP. In order to study this possibility we added the HMW fractions to C1q-depleted serum and followed C activity with and without C1q supplementation. Without C1q supplementation, there was no C activity in C1q depleted serum that was incubated with the HMW fractions, and activation occurred only after incubation with Zymosan, a known activator of the AP (Fig 4A). Upon supplementation of the C1q depleted serum with HMW fractions and commercial C1q, we found significantly increased C activity when the HMW fractions were from patients with detectable Ig-C5a (Fig 4B). As expected, in serum supplemented with C1q and Zymosan, the change in activity was minimal, while activation with an in-vitro preparation of aggregated IgG, a potent CP activator, was vast (Fig 4B).

### Identification of IgG in the HMW fractions

**Fig 5:**
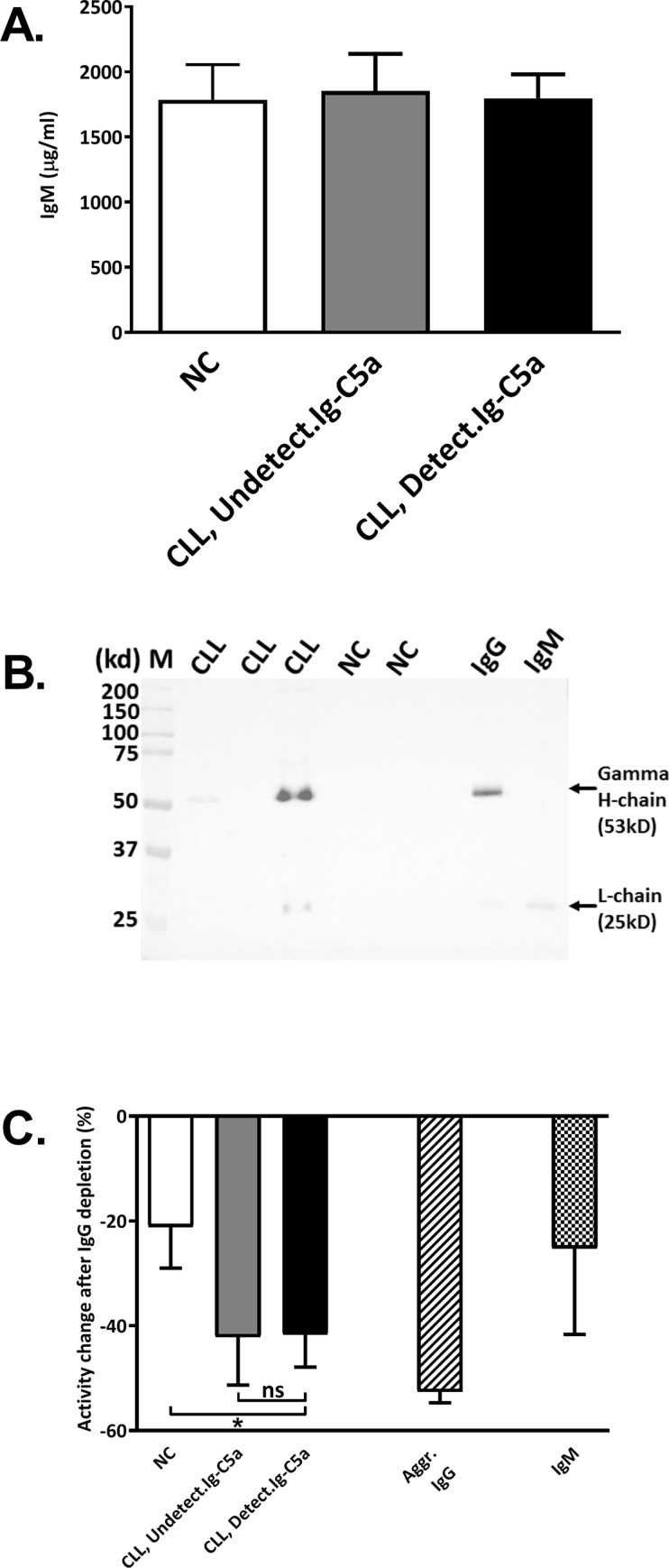
Identification of IgM and IgG in HMW fractions. Normal serum samples that were incubated with HMW fractions from NC and patients were used for measurements of IgM levels (**A**). IgG was followed in the HMW fractions from NC and patients by Western analysis (under reducing conditions) using anti-human IgG antibodies (**B**). Commercial IgG and IgM preparations were separated as positive and negative controls for the heavy chain signal. The gamma heavy chain and the light chain bands are indicated by arrows. IgG was depleted from the HMW fractions using a commercial kit, the HMW fractions with and without IgG depletion were used for activity analysis and the change in activity was calculated as percentage of the activity measured in the non-depleted fractions (**C**).

After showing that HMW fractions from patients with detectable Ig-C5a cause activation of the CP, we tried to identify more specifically the CP activator by studying IgG and IgM isotypes in the HMW fractions. The measurements of IgM levels in the HMW fractions showed similar IgM concentrations in all three subjects' groups (Fig 5A), thus ruling out IgM as the CP activator in the HMW fractions from patients with detectable Ig-C5a. The presence of IgG in these HMW fractions was demonstrated using Western blot analysis with anti-human IgG, which showed positive IgG signals in some CLL patients, but never in NC (Fig 5B). The presence of IgG and its role as the C activator in the HMW fractions from these patients was also demonstrated by the decrease in C activation after IgG depletion. IgG depletion from these fractions decreased their C activation ability by 45±6%, significantly more than in the HMW fractions collected from NC subjects (-21±8%), and similar to the decrease observed in the in vitro aggregated IgG (-52±2%, Fig 5C).

## Discussion

**Fig 6:**
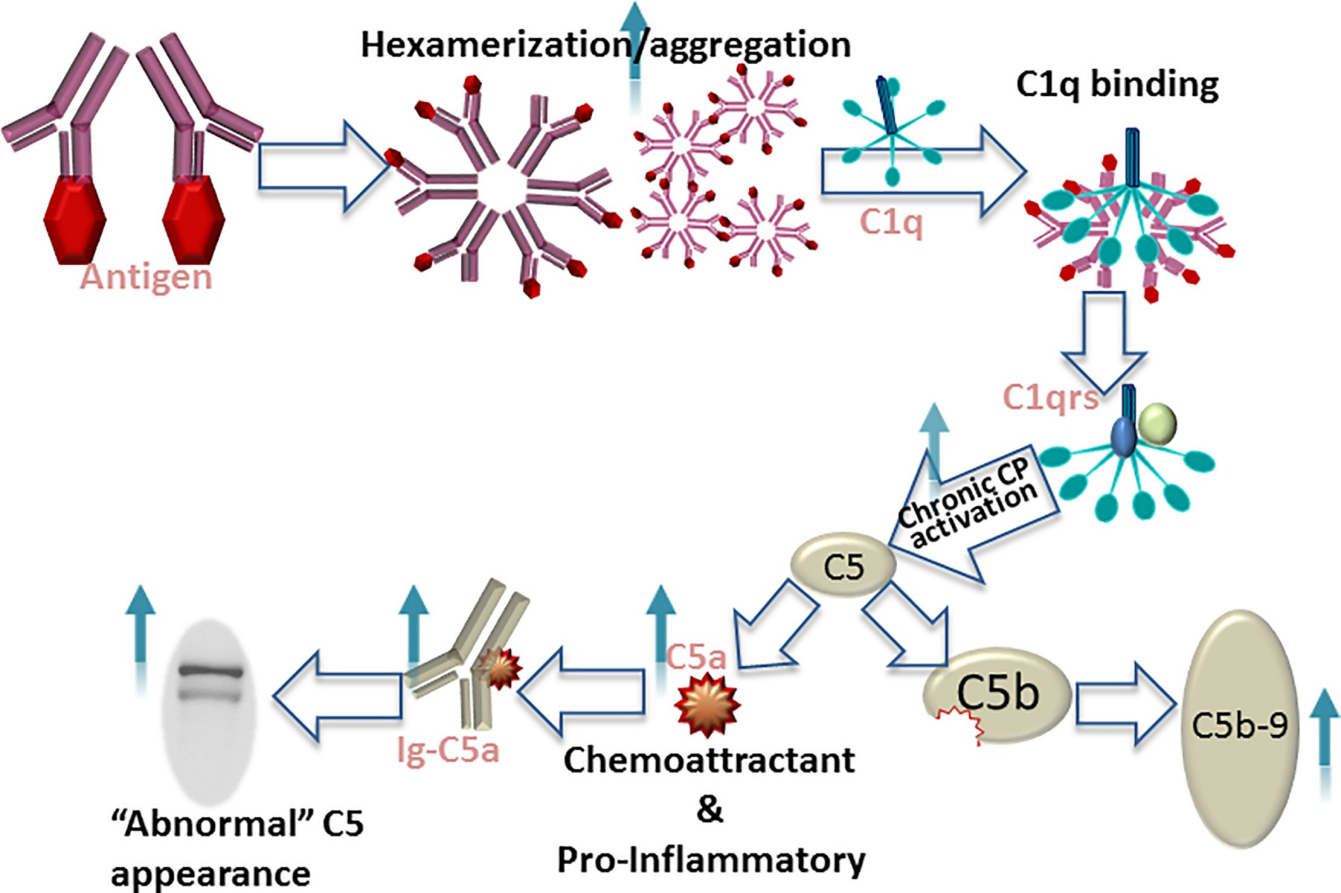
The mechanism of chronic CP activation in CLL. The CP is chronically activated due to the presence of IgG-hexamers/aggregates. IgG aggregates bind to the C1q component. This allows activation of the CP and generating C activation products such as C5a and C5b. C5b eventually forms the terminal C product C5b-9 (MAC) while C5a is bound to Ig molecules as part of a control mechanism aimed to reduce the potential hazards of C5a. The binding of C5a to Ig forms the Ig-C5a complex, described previously in CLL patients.

This study describes the presence of a cell-free C activator in plasma of some CLL patients. This C activator was identified as IgG-aggregates, an established activator of the CP, by several types of experiments. The presence of a CP activator in plasma of CLL patients can provide an explanation to the chronic CP activation and the CP exhaustion observed in some CLL patients. A schematic presentation of the events that occur in part of the CLL population is shown in Fig 6: the formation of cell-free IgG-hexamers (or aggregates of higher degree) chronically activates the C1q and the CP, resulting in increased levels of the C activation markers C5a and sC5b-9 as well as the Ig-C5a complex (observes previously as "abnormal" C5) [10].

The chronic CP activation is suggested by the increase in baseline levels of C activation markers such as C5b-9 and C5a ([10] and in this study) but also by studies which examined the plasma concentrations of C components. Early studies in CLL patients showed that the serum levels of at least one of the studied C components (C1q, C1r, C1s, C2, C3, C4, C5, C6, C7, C8, C9, Factor B and properdin) was reduced in 70% of the patients, as compared to NC subjects [7]. These findings were later supported by showing a significant deficiency in C2 and C4 concentrations in part of the CLL population, as well as a significant decrease in overall C activity [9,20]. These reported data support the chronic CP activation and the resulting CP exhaustion associated with low C2 levels, for example [9], which may limit the formation of C2a, a major component of the CP convertases, and thus decrease the activity of classical C5 and C3 convertases [10]. In agreement, a significant decrease in CP activity and in the activity of the classical C5 convertase was reported in some CLL patients [10].

In this study, both the CP and AP appeared to be chronically activated in part of the patient population. Yet, we examined the mechanisms related to chronic activation of the CP. There are several reasons for focusing on the CP: (1) the CP activity has great clinical importance in CLL; (2) the variety of activators for the CP is smaller compared to AP and thus the resolution of the mechanism for chronic CP activation was expected to be simpler; (3) the CP cascade lacks the self-enhancing loop of activation, which is found in the AP (involving the C3b molecule), therefore the observed chronic AP activation may be a secondary event, resulting from a primary CP activation; (4) the correlation data between sC5b-9 levels at baseline and after CP activation was more conclusive.

CP activity has great clinical importance in CLL as compromised C activity, combined with the known hypo-gammaglobulinemia in CLL, can increase patients' susceptibility to infections [7]. Low CP activity in CLL predicts short survival, and was the only factor from among various C factors that were investigated (including AP activity, levels of C1, C3, C4, factor B and C1-inhibitor, that significantly correlated with survival, [21]. CP activity is also clinically important for its role in C-dependent cytotoxicity (CDC) during immunotherapy. The therapeutic approach in CLL commonly includes chemotherapy and therapeutic monoclonal antibodies (mAbs, immunotherapy). The therapeutic mAbs mediate anti-tumor effects through several mechanisms: CDC, antibody-dependent cell-mediated cytotoxicity (ADCC), and phagocytosis. CDC is elicited via the CP, making CP activity/exhaustion an important aspect in immunotherapy outcomes.

In order for the CP to be chronically activated, one or more CP activators must be present in the circulation. The CP is activated mainly by HMW immunoglobulins, isotypes IgM and IgG, which bind to C1q via their Fc regions [22]. IgM exists in the circulation in a form of pentamer, containing a J chain, while IgG is normally found as a monomer. C1q, which consists of six collagen-like arms, has low affinity for immunoglobulin Fc in the monomeric form [22–24]. IgG binding and activation of C1q requires multimerization of IgG monomers into a hexamer structure [16,22]. IgG hexamers are formed after IgG binding to an antigen on a cell surface [16,17], and assembled via specific non-covalent interactions between the Fc of neighboring IgG monomers [22]. The hexamers are formed by interactions through the CH2–CH3 interface of the IgG monomers [16,24]. The hexamerized IgG complex is believed to form an optimal docking structure for C1 binding and CP activation. The ability to form IgG-hexamers depends on additional factors besides antigen binding, as can be deduced from the differences in hexamerization efficiency among IgG subtypes [16]. The hexamerization efficiency (IgG3 and IgG1 are superior to IgG2 and IgG4) originates from the amino acid sequence and structure of the heavy γ-chains [25,26]. These data were supported by mutation in the CH2–CH3 interface, (I235 in the CH2 and H433 in CH3) that individually affect C activation through C1q [16].

In this study we show that the serum of some CLL patients contains HMW CP activators that are not IgM. The identity of these HMW proteins as IgG-aggregates was confirmed by affinity binding assays and by Western blot analysis. The existence of serum IgG-aggregates, not bound to cell membranes (cell free), was not reported previously. Whether this hexamerization occurs initially on cell surfaces and is displaced off the cells, or as part of the humoral immunity, needs to be further investigated. Yet, this finding has great relevance for the CLL patients, since decreased CP activity (due to chronic C exhaustion) can affect both immunotherapy outcomes and morbidity in these patients.

The formation of the IgG-aggregates most probably involves interaction of IgG monomers with an antigen, bound or un-bound to cell surface, and can be related to the auto-immune characteristics that have been described in CLL, such as autoimmune hemolytic anemia, immune thrombocytopenia and other disturbances [27]. Autoimmune complications occur in up to 25% of CLL patients, primarily targeting the haematological lineages [28]. Moreover, CLL B-cells with unmutated IGHV gene, can present a highly polyreactive B-cell receptor (BCR) which recognizes auto-antigens [27,29]. Yet, in spite of numerous case reports showing nonhemic autoimmune disorders in CLL [27], there is no clear evidence or explanatory mechanism for these observations [27] and no understanding of the mechanisms that contribute to immune dysfunction or to autoimmune—therapy-related complications [28]. Clearly, further studies are required to resolve and clarify the autoimmune mechanisms related to CLL.

In conclusion, this study shows chronic CP activation, mediated by cell-free IgG-aggregates, as the cause of decreased CP activity in part of the CLL population. The significance of the findings is in its potential effect on immunotherapy outcomes, due to the compromised CP activity and CDC. Measurements of C activity markers offer a tool for presizing the immunotherapy regiments in CLL.

## Supporting information

S1 Raw images (PDF)
