# Supplementary material for: Cell-free IgG-aggregates in plasma of patients with chronic lymphocytic leukemia cause chronic activation of the classical complement pathway
Source: PLoS One. 2020 Mar 9;15(3):e0230033. doi: 10.1371/journal.pone.0230033 (PMC7062264; doi:10.1371/journal.pone.0230033)
Supplement: S1 Raw images — (PDF) [file pone.0230033.s001.pdf]

## The original gel used in Fig.5 panel B

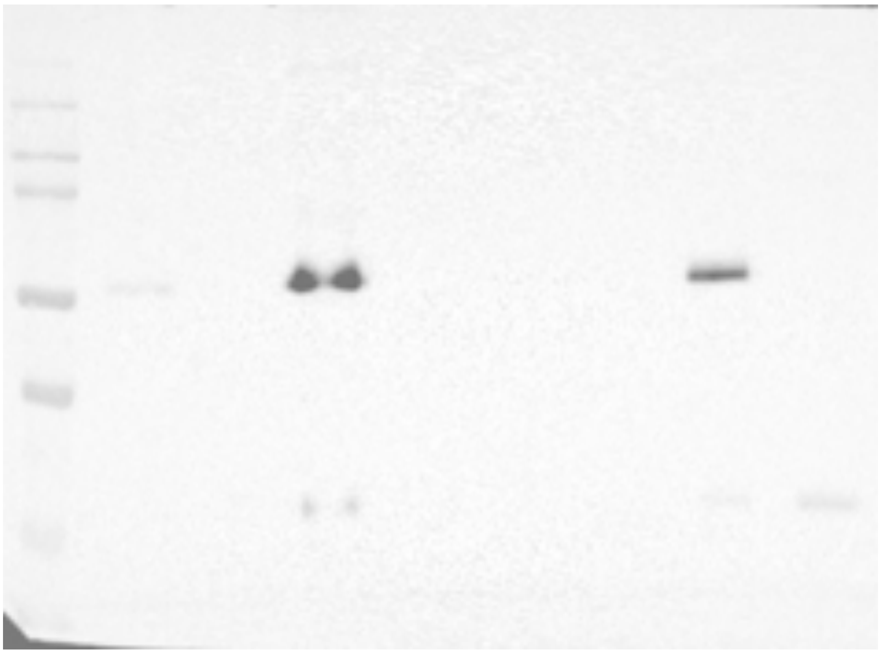


The gel was 10% Acrylamid, 1.5mm thick, samples were separated after denaturation. Running: 65min, 135v. Image was captured using the G-box imaging system. Samples loaded:

| Gel #150 lane | 1 | 2 | 3 | 4 | 5 | 6 | 7 | 8 | 9 | 10 |
|---|---|---|---|---|---|---|---|---|---|---|
| HMW Sample | M | Exp.109 CLL 3022 | Exp.109 CLL 3023 | Exp.109 CLL 3027 | Exp.109 NC 9703 | Exp.103 NC 12 | Exp.103 CLL 3011 | IgG | IgM | - |
| Total µg | | 10 | 10 | 10 | 10 | 10 | 3.8 | 0.2 | 0.2 | - |
| Denatur. | | + | + | + | + | + | + | + | + | - |

## The original gel used in Fig.6 & Graphical abstract. only lane 8 was used.

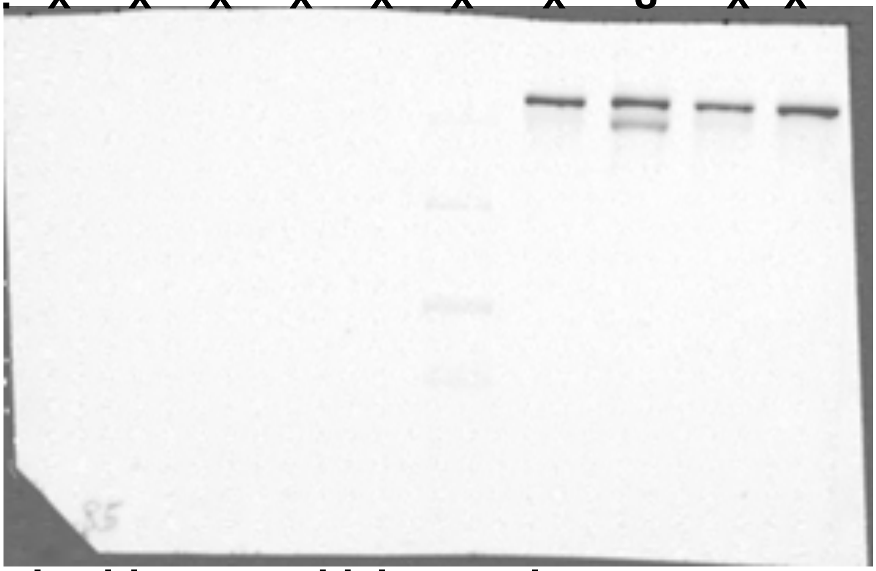


The gel was 8% Acrylamid, 1.5mm thick, samples were separated without denaturation. Running: 125min, 135v. Image was captured using the G-box imaging system. Samples loaded:

| Gel #085 | 1 | 2 | 3 | 4 | 5 | 6 | 7 | 8 | 9 | 10 |
|---|---|---|---|---|---|---|---|---|---|---|
| Sample # | | | | | | M | CLL 3002 | CLL 9729K | CLL 3014Z | CLL 3020Z |
| Vol (ul) | | | | | | 2.5 | 15 | 15 | 15 | 15 |
| | | | | | | | Serx100 | Serx100 | Serx100 | Serx100 |
